# Soft Robotics in Minimally Invasive Surgery

**DOI:** 10.1089/soro.2018.0136

**Published:** 2019-08-02

**Authors:** Mark Runciman, Ara Darzi, George P. Mylonas

**Affiliations:** ^1^ Human-Centred Automation, Robotics and Monitoring in Surgery (HARMS) Lab, Department of Surgery and Cancer, Imperial College London, London, United Kingdom.; ^2^ Department of Surgery and Cancer, Imperial College London, London, United Kingdom.

**Keywords:** medical robotics, minimally invasive surgery, soft robots

## Abstract

Soft robotic devices have desirable traits for applications in minimally invasive surgery (MIS), but many interdisciplinary challenges remain unsolved. To understand current technologies, we carried out a keyword search using the Web of Science and Scopus databases, applied inclusion and exclusion criteria, and compared several characteristics of the soft robotic devices for MIS in the resulting articles. There was low diversity in the device designs and a wide-ranging level of detail regarding their capabilities. We propose a standardized comparison methodology to characterize soft robotics for various MIS applications, which will aid designers producing the next generation of devices.

## Introduction

Minimally invasive surgery (MIS) involves the use of long rigid or flexible surgical instruments that are inserted into the body through small incisions or natural orifices, in contrast to open surgery where large incisions are used to access the target anatomy directly. The goal of MIS is to complete a surgical procedure as safely and quickly as possible, while minimizing damage to peripheral tissue. MIS is being used with increasing frequency as an alternative to open surgery because of the improvements it can bring to patient safety, cosmesis, recovery time, shorter hospital stay, fewer postoperative complications, and pain.^1^ This review details a literature search targeted at articles describing novel soft robotic devices for MIS.

Central to MIS is the field of endoscopy; the process of viewing the inside of the body by directly inserting an optical device into the area of interest. The optical device is called an endoscope and several different types exist. Today, an endoscope commonly refers to a long flexible tube approximately 1.5–2 m in length equipped with a high-resolution camera and a light source at its tip. The tip can be actively steered by means of two thumb-controlled dials at its proximal end. Typically, there are working channels along the endoscope's length to supply air and water, and through which small, flexible instruments can be introduced for performing basic therapeutic procedures. Flexible endoscopes of this description are used for visualization of the upper gastrointestinal (GI) tract (gastroscope) and lower GI tract (colonoscope). However, rigid endoscopes of varying length and diameter are also used in many applications, for example, to visualize the abdomen (laparoscope), brain, (neuroendoscope), joints (arthroscope), and esophagus (esophagoscope). There are a wide range of endoscopic procedures involving either diagnosis or therapy on many parts of the body. Flexible and rigid endoscopes can vary in diameter and length depending on the application and the patient.

Surgical tools allow surgeons to grasp, dissect, remove, and suture tissue inside the body.^2^ A common example of MIS is endoscopic surgery for abdominal procedures, where a laparoscope and two or three long, rigid surgical tools of typical diameter around 5 mm are introduced into the abdomen through multiple individual small incisions. Several approaches have been developed to make MIS even less invasive and to enable new procedures that are impossible with traditional open surgery. One of these improved approaches is single-incision laparoscopic surgery, which involves inserting not only a laparoscope but also two rigid instruments through a single larger incision in the abdomen, preferably at the umbilicus, therefore reducing the number of incisions, but increasing the difficulty of the procedure. Natural orifice transluminal endoscopic surgery (NOTES) is a technique in which the abdomen is accessed using a long, flexible endoscope inserted through the mouth, anus, or vagina, and offers the benefit of avoiding abdominal incisions entirely.^3^ Instead, NOTES is performed through internal incisions that allow the endoscope to cross between tubular structures within the body, known as lumen, to adjacent cavities. MIS can also be performed on the brain by removing part of the skull and placing a port, through which a neuroendoscope and surgical instruments are passed to gain access to target tissue deep in the brain.^4^

MIS is characterized by small, easily deformable, dynamically changing, and unstructured workspaces, poor visibility with few visual markers for orientation, and the use of long, narrow instruments. Long, rigid instruments used in some forms of MIS suffer from the fulcrum effect, caused by the point of insertion of the instrument into the body, acting as a point of rotation that inverts the surgeon's movements and can amplify hand tremor, making the instruments more difficult to use.^5^ In current robotic MIS approaches, a surgeon controls a rigid robotic device that in turn controls the motion of the modified surgical instruments. The forces exerted at the tip of manually operated laparoscopic instruments can range between 0.1 and 10 N,^6^ so designers of robotic systems aim to achieve similar performance. In addition, robotic systems deliver precision, stability, motion scaling, and other benefits, but are unable to navigate tortuous paths due to their inflexibility and sometimes their large size, meaning they cannot provide access to all target anatomy. Flexible endoscopes and instruments are therefore used when the surgical site cannot be reached by rigid devices and, if flexible devices would be ineffective, open surgery may be the only option. Robotic systems with multiple instruments are also affected by instrument clashing,^7^ which makes manipulating the instruments more complex due to their overlapping workspaces. Using some robotic systems can also present difficulties with changing instruments during a procedure.

Instruments that are difficult to use result in lengthy procedures and high risk of causing unwanted damage to the patient.^8^ Furthermore, years of training are sometimes required to become an expert in their use. Patient pain is often caused as a result of the instruments deforming or perforating the tissue surrounding them, which can be caused by using endoscopic instruments that are too stiff.^9^ Damage or pain to the patient can also be caused when using flexible devices, and an example of this is looping of the colon during colonoscopy.^10^ In addition, problems still remain with positioning, dexterity, force exertion, and visualization when using flexible instruments and endoscopes.^11^ Research into soft robotics aims to bring together the controllability of rigid robotics, the access capabilities of flexible instruments, and the safety of soft materials by solving these problems.

Soft robotics focuses on using soft, compliant materials to construct robotic devices. Due to the materials they are made from, soft robots are ideal for dealing with unstructured environments or interacting with humans because they can deform around their environment.^12^ This differs from the traditional robot design approach of using rigid materials for both robot links and joints and is very well suited to medical applications, where eliminating patient trauma and pain are highly important.^13^ The challenges faced in MIS make compliance, variable stiffness, and safety some of the most important design criteria,^14^ and the field of soft robotics is well placed to meet these demands. In the authors' experience, soft materials achieve high patient acceptability in comparison with robotic devices made from metallic or other rigid materials. A colon cancer patient representation group also found an unintimidating appearance to be more important than the footprint. Clinicians specializing in applications on the GI tract and who are familiar with its delicate mechanical properties also expressed a preference for soft devices that would be less likely to cause patient pain and trauma.

Unfortunately, there are many trade-offs in return for the increase to patient safety, including low force exertion, poor controllability, and a lack of sensing capabilities, as for example discussed in Hughes *et al*.^15^ Simulation of soft robots is difficult and computationally expensive because compliant materials exhibit nonlinear responses to strain and soft devices have many degrees of freedom, which hinders the ability to design soft robots predictably.^16^ Soft robots can achieve large changes in volume, shape, and stiffness, which are impossible for conventional ones and which give them a unique advantage. Harnessing these capabilities and tackling the problems with low force exertion and controllability will deliver the next-generation instruments for MIS.

An advantage of soft materials is that they are often economical, readily available, and easy to handle; the most common example being the large range of elastomers used in much of the current research. By using economical soft materials and new manufacturing techniques, it is also becoming possible to develop disposable, patient-specific, low-cost, and rapidly manufactured robotic devices for MIS. Being more affordable than traditional robotic systems, soft robotic devices have the potential to become widely available^17^ and this makes them a candidate for frugal design approaches that could have high impact. Further advancements in materials and manufacturing will confirm the potential for patient-specific soft robotics in the future. However, there may be additional regulatory requirements for customizable medical devices. Specifically, manufacturers must ensure that each customized device meets the appropriate quality and safety requirements; hence, reliable manufacture is highly important.

The objective of this review is to provide an overview of the soft robotic devices that are under development in the field of MIS so that designers may more easily identify new ways of overcoming the numerous challenges that are currently faced.

In the next section, the methodology used to carry out the literature search is reported, followed by a description of the comparison process and the results of the literature search. The application areas of the devices include endoscopic procedures for both diagnostic and therapeutic purposes. Throughout the rest of this review, the working principles, materials, manufacturing, actuation, variable stiffness, locomotion, and sensing methods found on the selected soft robotic devices are described in more detail. This will highlight the challenges across many disciplines that have to be considered and the lack of a standard method of comparison for soft robotic devices in MIS.

## Literature Search

A keyword search was performed on Web of Science and Scopus for articles describing soft robotic devices designed for use in surgery or MIS procedures. The keyword searches used in the selected databases with the appropriate Boolean operators and syntax are summarized in Table 1. The reference lists of these articles and other similar articles were also used as a source for a small proportion of additional search results. The inclusion and exclusion criteria described below were then applied and duplicate articles removed. This yielded 35 unique articles, each detailing a soft robotic device for use in minimally invasive applications.

**Table 1.** Keyword Search Terms and Results

*Database*	*Keyword search terms*	*No. of results*	*Date*
Web of Science	TS = (“soft robot^*^”) AND TS = (MIS OR “minimal^*^ invasiv^*^” OR endoscop^*^)	93	10/12/18
Scopus	TITLE-ABS-KEY (“soft robot^*^”) AND (TITLE-ABS-KEY (mis OR “minimal^*^ invasiv^*^”) OR TITLE-ABS-KEY (endoscop^*^))	88	10/12/18

### Inclusion criteria

- The article described the design and/or manufacture of a robotic device.
- The device described was made of soft materials and relied on soft robotic principles.
- The main application for the device was surgery, MIS, endoscopy, laparoscopy, bronchoscopy, colonoscopy, or catheterization.
- The article was written in English.
- The article was accessible by the author at the time of the search.

### Exclusion criteria

- The device described was not designed to be used in MIS procedures.
- The device was not compliant, or it contained too many rigid parts to be easily compressed.

**FIG. 1.**
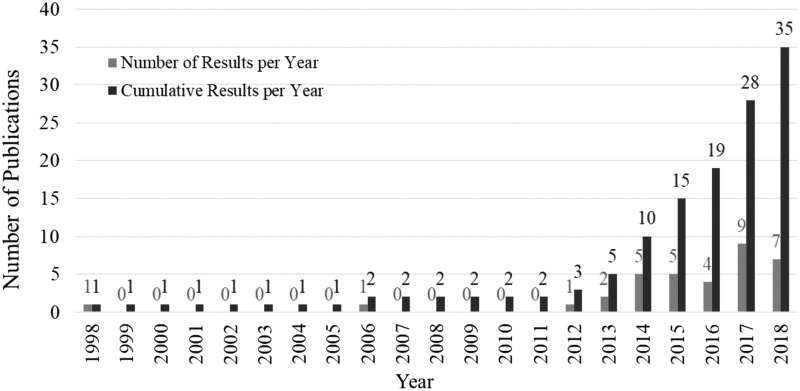
Number of results and cumulative results per year.

There has been a growth in interest each year since around 2013, which can be seen by the number and cumulative total of results plotted in Figure 1. This was possibly influenced by the STIFF-FLOP surgical manipulator project^18^ that started in 2012.

## Comparison Framework

Of the soft robotic devices in the articles remaining at the end of the literature search procedure, the main characteristics were noted. These characteristics are summarized in Table 2. This was done to more easily compare distinct devices and highlight differences in the various categories. In the next section, the result of the device comparison process is shown.

**Table 2.** Characteristics Used for Comparison Framework

*Characteristic*	*Description*
Application	The application for which the device was designed. These include MIS, which may require incision, and endoscopy, which uses natural orifices and manipulation for devices that were designed to grip.
Working Principle	Fundamental structure of robot that was designed. Examples include the following: continuum, serial, and peristaltic.
Dimensions	Characteristic dimensions of the device.
Materials	Materials used in the device construction focus on soft materials.
Manufacturing Methods	Main method of fabrication, whether it requires single or multiple processes, manual or automatic assembly.
Actuation Methods	All actuation and stiffening methods featured on the device.
Locomotion	Method of positioning the robot, whether it is manually or robotically positioned or self-propelled.
Sensors	All proprioceptive or diagnostic sensors on the device.
Force exertion	Results of testing carried out on each device. Varies based on type of robot.
Bending, twisting, elongation, expansion	The extent of each motion as appropriate. Varies based on type of robot.
Variable Stiffness Mechanisms	Describing the capability to control the stiffness. Varies based on type of robot.
Instrumentation/instrumentation channels	Description of embedded instrumentation or number of channels available for instruments.

MIS, minimally invasive surgery.

## Results

The articles yielded by the literature search were compared using the framework described in the previous section and the results can be found in Table 3.

**Table 3.** Results of Literature Search After Application of Comparison Framework

*Ref.*	*Application*	*Working principle*	*Dimensions/mm*	*Materials*	*Manufacture and assembly*	*Actuation*	*Locomotion/positioning*	*Sensors/feedback*	*Force exertion*	*Bending, twisting, elongation*	*Variable stiffness*	*Instrument channels*
^19^	MIS (cardiothoracic)	Continuum	320 mm length, 30–10 mm OD	Elastomer	Molding	Cable driven	Manual	Four optical fibers		Reduction of distance error		One channel
^20^	MIS (cardiothoracic)	Continuum	300 mm length, 9–30 mm OD	Elastomer	Molding	Cable driven	Robotic	Magnetic guidance				One for 5 mm endoscope, one for instrument
^21^	Endoscopy (support sleeve)	Continuum	400 mm length, 22 mm OD	PET, Latex	Laser cutting, fiber reinforcement, sewing, interweaving, molding	Cable driven, layer jamming	Manual	None	2 N payload	Some elongation		15 mm Channel
^22^	MIS	Continuum	200 mm length, 23 mm OD	Latex, polyester	Sewing	Cable driven, pneumatic	Manual	None		180° Max bending	2.1875 Times axial stiffening, 106.67–233.34 N/m at 7.5–15 kPa	None
^23^	MIS	Continuum	32 mm OD, 50 mm length	Elastomer	Multistage molding, fiber reinforcement	Hydraulic	Manual	Hydraulic pressure				One channel
^24^	Endoscopy	Continuum	15 mm OD, 100 mm length	Elastomer	Multistage molding, fiber reinforcement/extrusion	Hydraulic	Manual	Pressure sensors, image processing		110° Max bending at 50 kPa		Diathermy, biopsy channel, camera
^25^	Bronchoscopy	Continuum	7 mm OD, 32 mm length	Elastomer	3D printing, molding	Pneumatic	Manual	None		61.5° Twist at 300 kPa, 59° and 35° max bend for respective chamber at 350 kPa		None
^26^	Endoscopy	Continuum	1.66 mm OD, 12 mm length	Elastomer	Molding	Pneumatic	Manual	Pressure	Low	160° at 280 kPa, 45° at 280 kPa with camera		CMOS camera
^27^	MIS, prosthetics	Continuum	18 mm OD, 85 mm length	Elastomer	Molding, 3D printing	Pneumatic	Manual	None		270° at 45 kPa, 23° twist at 40 kPa		None
^28^	MIS	Continuum	6 mm OD	Elastomer	Molding, 3D printing, fiber reinforcement, adhesion	Pneumatic	Manual	Electromagnetic tracking		180° Bend, 5 mm bend radius		One channel
^29^	MIS	Continuum	13 mm OD, 93 mm length	Elastomer	Molding, fiber reinforcement	Pneumatic	Manual	Electromagnetic tracking	0.96 N max contact force	>150° Omnidirectional		None
^30^	Optical Biopsy	Continuum	<10 mm Width	Elastomer	Molding, lithography, vacuum ultraviolet-assisted bonding	Pneumatic	Manual	Two waveguides for fluorescence imaging		120° at 120 kPa		None
^31^	MIS	Continuum	1 mm OD, <20 mm length	Elastomer	Molding, micromachining	Pneumatic	Manual	None		7.14 mm bend radius at 100 kPa	Stiffness changes with increasing pressure	None
^32^	MIS	Continuum	25 mm OD, 65 mm length	Elastomer	Multistage molding	Pneumatic	Manual	Electromagnetic tracking for testing	4.6 N at 0.3 bar	90° with different working pressures		None
^33^	MIS	Continuum	84 mm length, 14 mm OD	Elastomer	Multistage molding, adhesion	Pneumatic	Manual	None	9 N at tip	142.4° Max at 85.5 kPa		None
^34^	Manipulation (microscale)	Continuum	3 × 25 mm rectangle	Elastomer	Multistage spin coating	Pneumatic	Manual	None	Low torque	Up to 210° bending		None
^35^	MIS	Continuum	70 mm OD semi-circle, 140 mm length	Elastomer, leather	Molding, sewing, 3D printing	Pneumatic, granular jamming (passive)	Manual	None	∼8 N at ∼100 kPa	10 cm Bend radius	∼4k mNm/rad	None
^36^	Endoscopy	Continuum	—	Elastomer	3D printing, molding	Pneumatic, SMA	Manual	None				One channel
^37^	Catheterization	Continuum	170 mm Long, 4 mm OD	Elastomer	Molding, CNC machining	SMA	Manual	None		270° Twist at 1.25 A		None
^38^	MIS	Continuum (modular)	20 mm OD, 83.5 mm length	Thermoplastic elastomer	3D printing	Cable driven	Manual	Electromagnetic tracking for testing	1.5 N generated force	∼100° Asymmetric bending and twisting		Manipulator on tip
^39^	MIS	Continuum (modular)	23 mm Ø, 47 mm length	Elastomer, PET	Molding	Cable driven, pneumatic	Manual	Pressure and tension			Stiffness change of up to 94%, from 130 to 250 N/m	One channel, large
^40^	Catheterization	Continuum (modular)	3 mm OD, 10 mm valve length, 25 mm length	Elastomer, polymer	Microstereolithography, molding	Hydraulic	Manual	Pressure, no bending angle sensing		170° Max		0.5 mm Channel
^41^	MIS	Continuum (modular)	14.5 mm OD, 50 mm length	Elastomer	Molding, 3D printing, fiber reinforcement	Pneumatic, granular jamming	Manual	Electromagnetic tracking for testing		171° at 1.5 bar		3 mm Channel
^42^	MIS	Continuum (modular)	14.3 mm OD, 60 mm length	Elastomer	Molding, 3D printing, fiber reinforcement	Pneumatic, granular jamming	Robotic	Electromagnetic tracking				4 mm Channel
^43^	MIS	Continuum (modular)	10 mm OD, 30 mm length	Elastomer	Multistage molding	Pneumatic/hydraulic	Manual	Electromagnetic tracking for testing				One channel
^44^	MIS	Continuum (modular)	14.5 mm OD, 50 mm length	Elastomer	Molding, fiber reinforcement	Pneumatic, granular jamming	Robotic	None				One channel
^45^	Endoscopy	Continuum (modular)	32 mm Ø, 50 mm length	Elastomer, PET	Multistage molding, fiber reinforcement	Pneumatic, granular jamming	Manual	None	47.1 N max		46% Stiff variation	Two channels
^46^	Endoscopy	Continuum, peristaltic	35 mm max Ø, 500 mm length	PET	Heat crimping, 3D printing	Cable driven	Self-propelled	Hall sensors for tendon length		90° Max, 25 mm length change		6 mm Camera
^47^	Endoscopy	Continuum, peristaltic	OD 5.5 mm, 45 mm length	Elastomer, steel, polymer	Multistage assembly	SMA	Self-propelled	None		90° Max, 19 mm radius		CCD image sensor
^48^	Manipulation (microscale)	Gripper	3–5 mm Width	Elastomer	Molding, 3D printing	Pneumatic	Manual	None				None
^49^	Drug delivery, manipulation (microscale)	Mobile gripper	3.7 mm Length, 1.5 mm width, 0.185 mm height	Elastomer, neodymium-iron-boron micro particles	Molding, laser cutting, magnetization	Magnetic	Magnetic positioning	None				None
^50^	Manipulation (microscale)	Mobile gripper	5 mm Ø	Hydrogel	Photolithography	Magnetic, thermal	Magnetic positioning	Haptic assistance—camera		10 s Open/close transition		None
^51^	Insertion aid (colon)	Peristaltic	16 mm OD, 700 mm length	Elastomer	Injection molding	Pneumatic	Manual	None				N/A
^52^	Endoscopy	Peristaltic	3 mm OD, 250 mm length	Elastomer/PVC resin	Molding/extrusion	Pneumatic	Self-propelled	None	Low			One channel
^53^	Endoscopy	Peristaltic (modular)	8 mm OD, 7 mm ID	Elastomer	Molding, 3D printing, fiber reinforcement	Pneumatic	Self-propelled	None		Extension, expansion, twist		Instrument on tip

Ø, Diameter; 3D, three dimensional; ID, inner diameter; OD, outer diameter; PET, polyethylene terephthalate; SMA, shape memory alloy.

### Working principles

There are many different ways to design robotics systems and this section describes the working principles of some of the soft robots that resulted from the literature search.

#### Continuum robots

**FIG. 2.**
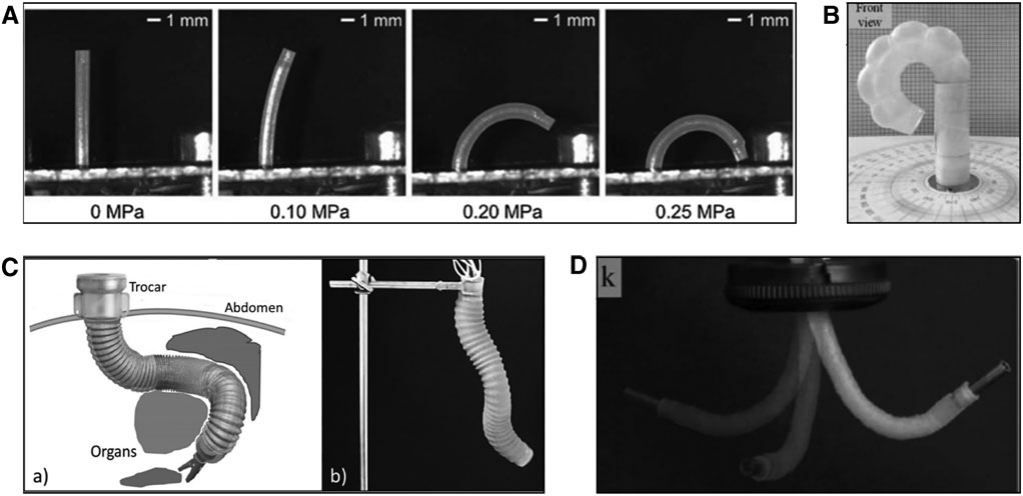
Examples of continuum robots for MIS: **(A)** a single-chamber pneumatic microactuator,^31^ **(B)** a three-chambered pneumatic module,^27^ **(C)** the three-module STIFF-FLOP manipulator,^88^ **(D)** a miniature three-chambered manipulator with outer diameter of 6 mm.^28^ MIS, minimally invasive surgery. Figure panels were reprinted with permission from their respective references.

Continuum robots are robotic devices whose bodies do not contain single rigid links or joints, but are able to bend continuously and can be considered to have an infinite number of joints. Octopus tentacles are a source of inspiration for soft continuum robots as they can achieve highly dexterous control, bending, and stiffening behavior, despite consisting of purely soft tissue. Many continuum robot designs are also modular or composed of several base units that can be controlled separately, as opposed to a single unit. Figure 2 shows some examples of different styles of continuum robot. Modularity can have benefits when different parts of the robot are required to have different qualities, for example, the middle section of an arm is required to exert force or remain rigid, while the tip exhibits compliance.^45^

Continuum robots seem to be an appropriate choice for MIS applications because they require only one entry point and can achieve large bending angles, permitting exploration of body cavities, which is impossible with rigid instruments. However, continuum robots also require a base or support structure and the ability to exert force diminishes along their length, which produces a trade-off between actuation and explorative capabilities. This style of robot was the most common among the 32 results of the literature search, with 18 single module continuum robots,^19–34^ 6 modular continuum robots,^38–43^ and 2 robots capable of both peristaltic crawling and continuum motion.^46,47^

#### Peristaltic robots

Peristaltic robots are self-propelled devices that often take inspiration from the likes of earthworms, inchworms, and snakes. These three models differ in certain ways, but all of them depend on anisotropic friction to achieve locomotion.^54^ Only inchworm- and earthworm-style peristaltic motion were observed in the results of the literature review and they are described here.

In earthworms, the radii of several segments of its tube-like body are reduced at the same time as their lengths are increased, while other segments behind them expand their radii and shorten their length. This pattern of contraction/elongation and expansion/shortening moves along the body of the worm in the opposite direction to its motion. The effect of this is to provide anchor points behind the elongating sections such that the friction in the forward direction is less than that in the backward direction. This form of peristaltic crawling was the most popular among the self-propelling robots in the results, used in three out of four examples.^51,53,55^ The method in Suzumori *et al*.^51^ is slightly different from the others, as an elastomer sheath wrapped around a colonoscope was produced to help with scope advancement by generating travelling deformation waves, in contrast to the self-propelling colonoscope replacements in Connolly *et al*.^53^ and Takeshima and Takayama.^55^

The mechanism for inchworm motion is similar, except that anchor points are found only at the head and tail, while the body is contracted and elongated. An example of inchworm-style motion was implemented in a self-propelled endoscope that makes use of a cable-driven actuation mechanism,^46^ which anchors its head or tail by pressing into the colon wall, and then advances by releasing cable tension in the center of its body, which elongates using passive spring action.

Soft peristaltic robots provide the potential to provide access without stretching the colon and causing damage or pain to the patient as current techniques do. Furthermore, self-propelling devices offer a step toward the automation of routine procedures. Effortlessly advancing through the colon would allow more attention to be devoted to diagnosis.

#### Serial robots

Serial robots consist of several prismatic or rotational joints that are coupled together by links, often to form the well-known robot arm structure. This type of robot is mentioned in this study because it is usually associated with traditional robotics constructed from rigid materials; however, a soft serial robot consisting of pneumatic stiffened links and pneumatically actuated joints has been developed for safe interactions between people and industrial robots, achieving positioning error of <1 mm.^56^ Soft pneumatic joints have also been developed, which can produce both linear and rotational motion for use in grippers, robotic arms and even mobile robots.^57^ In relation to medical applications, a pneumatically actuated serial mechanism was attached to the end of an endoscope in Russo *et al*.^58^ This mechanism integrates soft fluidic actuators into a rigid resin structure. Some grippers rely on serial mechanisms, such as that described in Pacchierotti *et al*.,^50^ where straight, but soft fingers on a miniature starfish-like robot are actuated at a soft joint.

A limitation of soft serial-type robots is that serial robots rely on discrete links to transfer force, so using deformable materials or variable stiffness mechanisms leads to low force exertion or large size. Furthermore, accurate control is achieved in traditional robots that use rigid components because the link lengths, joint rotations, and joint extensions are known or can be easily measured, whereas the same cannot be said for their soft counterparts. Their low controllability may be the main reason that no soft robot arms for MIS were observed in the literature search, coupled with the fact that the manufacture of a soft robot with several discrete links and joints would be more complex than the manufacture of a continuum robot.^1^

Hyper-redundant serial robots are composed of a large number of joints and have similar benefits to continuum robots, but also suffer similar trade-offs. Serial robots with high degrees of freedom (DOFs) are capable of assuming complex poses that could allow them to navigate tortuous paths and inspect complex shaped objects; however, force exertion diminishes with increasing length. In this way, they are apparently suitable to MIS applications, however, no articles describing hyper-redundant soft serial robots were yielded by the literature search. Complex manufacture and control appear to be the main challenges to face, but this may be an interesting target for research if appropriate manufacturing methods are developed.

### Materials

Soft materials provide a valuable resource for designing atraumatic medical devices for use within the body because they can deform before causing damage. Soft materials include elastomers, such as polydimethylsiloxane (PDMS) and polyurethane; thin layers of plastics, such as polyethylene (PE), polyethylene terephthalate (PET), or polypropylene (PP); and even metals that exhibit superelasticity and that have been manufactured to have very low cross-sectional area, such as Nickel Titanium (NiTi, Nitinol). Elastomers were used in 31 devices in the results of the literature review, plastics in 8, latex in 2 devices, and hydrogel and leather used in just one each. Three devices used NiTi wire as an actuator. One of the benefits of using elastomers and plastics in particular is their low cost and ready availability. As a result of using soft materials, it is likely that a soft device is magnetic resonance imaging compatible, which designers can capitalize on.

In some cases, it was unclear whether a device should be included in this review based on the rigidity of the materials used. For example, the continuum robots designed in Li and Du^59^ and Li *et al*.^60^ rely upon a series of vertebrae as the main support structure, which makes them highly flexible until some bending limit is reached and the vertebrae lock together. These may at first appear to be soft robots as they use soft materials and use actuation mechanisms commonly seen in the soft robotics field, such as cable-driven mechanisms. However, not all of the materials throughout the devices exhibit compliance and, therefore, still pose the threat of causing unwanted harm within the body due to their rigidity. Compliance, *k^−1^*, is related to the length, *L*, elastic modulus, *E*, and the cross-sectional area, *A*, of a material, as shown in Equation (1).

\begin{align*}
k^ { - \it { 1 } } = \frac { L }  { { E \cdot A } } \tag { 1 } 
\end{align*}

A device that is constructed from materials whose elastic moduli are orders of magnitude greater than human tissues can still therefore be classified as a soft robot if the materials' cross-sectional areas are small.^61^ Many elastomers are highly compliant due to their low Young's moduli, making them a popular choice for interaction with the patient in noninvasive medical applications.

A robot's stiffness, force exertion, and bending/twisting/elongation/expansion can all be dependent on the elastomer used because the deformable bodies of elastomer-based robots often contain the chambers used for fluidic actuation. Extra care must therefore be taken when selecting elastomeric materials, due to their influence on many aspects of a device's functionality.

The development of soft analogues for more complex, currently rigid mechanisms is another challenge. Given that fluidic actuation is a popular actuation method, soft valves are of particular interest. Soft valves inspired by the three-flap, cone-shaped mitral valve in the heart were manufactured by silicone molding in Gilbertson *et al*.,^62^ as an enabling technology for an entirely soft, self-propelling catheter. Compliant valves that controlled flow by using the electrorheological principle were three-dimensional (3D) printed in Zatopa *et al*.,^63^ using liquid metal eutectic gallium-indium-tin as the electrodes. The hydraulic valve could withstand over 250 kPa of pressure when activated at a voltage of 5 kV, but this varied when the valve was put under strain. This is just one example of how using soft materials bring new challenges that have not been encountered with traditional materials; hence, making soft replacements for traditionally rigid components remains a challenge.

### Manufacturing methods

There are a variety of manufacturing methods that can be used to make soft robotic devices. A brief summary of the most common methods is made in this section, followed by an analysis of the manufacturing methods that were used to produce the devices yielded in the keyword search. Novel manufacturing methods will play a key role in developing patient-specific devices.

Time, cost, repeatability, reliability, adaptability, expandability, quality, size, feature size, equipment, and compatible materials are all factors that need to be considered when choosing manufacturing methods. Another of the challenges with patient-specific devices is to ensure the manufacturing quality of each individual device. Making sure the performance, reliability, and safety of multiple units that have each been adapted for particular patients and particular medical operations present a substantial hurdle using current approaches. Furthermore, the ability to demonstrate the safety of all variations of a general device design within a certain range of dimensions, applications, and target anatomy may also present issues with certification, which will also have to develop in conjunction with the patient-specific surgical technology that will be seen in the near future.

Manufacturing approaches using rigid plastics have been developed, which aim to make patient-specific, disposable devices feasible in terms of manufacture time and cost by using additive manufacturing.^64,65^ However, there are still many limitations to producing robotic devices made of soft materials quickly and cheaply, while maintaining the high level of performance necessary in MIS applications. Although the materials used in elastomer molding are economical, the time required for manually carrying out additional manufacturing processes (such as fiber reinforcement, CNC machining, or 3D printing) and additional assembly (such as adhesion, placement of a sheath, or connection of multiple modules) often hinders rapid manufacture. High manufacturing times or high costs, due to complex procedures, equipment, or materials, prohibit the development of disposable, single-use, or patient-specific devices. A summary of manufacturing processes that can be used to make soft robotic devices is found in Table 4.

**Table 4.** Manufacturing Process and Their Advantages and Disadvantages

*Manufacture Process*	*Description*	*Advantages*	*Disadvantages*
Molding	A mold is manufactured and filled with elastomer that is left to cure.	Simple process for simple parts; molds can be manufactured cheaply and relatively quickly, often using additive manufacturing; repeatable (based on mold manufacture); expandable.	Limited material selection; defects such as bubbles can form in the elastomer body; often requires additional parts/materials to reinforce or prevent large deformations; lower limit on size; lower limit on feature size; adding additional features can make manufacture complex.
Extrusion	Material is forced through a die to produce a given cross section.	Large-scale production; long parts are possible; repeatable; forms internal lumen.	High equipment costs; cross-section is constant along the length; cross-sectional dies are not easily adapted; feature size limited to.
3D printing elastomers	Devices are printed one layer at a time using thermoplastic elastomer or with a curing process.	Highly adaptable; repeatable; expandable; range of design sizes.	Support material required, can limit size as material deforms under own weight; time-consuming.
Shape deposition	Multiple molds and steps are used to mold varying materials in complex shapes.	Allows for embedding of components into a part; can combine different materials; allows convex features.	Multistep process; time-consuming.
Soft lithography	Begin with bulk soft material substrate and either remove or deposit new material where the substrate has been exposed to radiation.	Very small feature sizes possible, down to ∼500 μm; repeatable process.	Upper limit on device size; time consuming; difficult to incorporate additional functionality onto small devices.
Micro stereo lithography	A series of cross-sectional layers are built up one at a time by radiating material at the base.	Repeatable process; adaptable.	Upper limit on device size; difficult to incorporate additional functionality onto small devices.
Spin coating	A substrate is rapidly rotated after application of a coating fluid, depositing a thin film on the substrate after a given time due to the centrifugal effect.	Possible to produce very thin films; quick process; repeatable	Multistage process, punctuated by curing processes and actuation fluid channel placement; upper limit on size; difficult to incorporate additional functionality onto small devices.

**FIG. 3.**
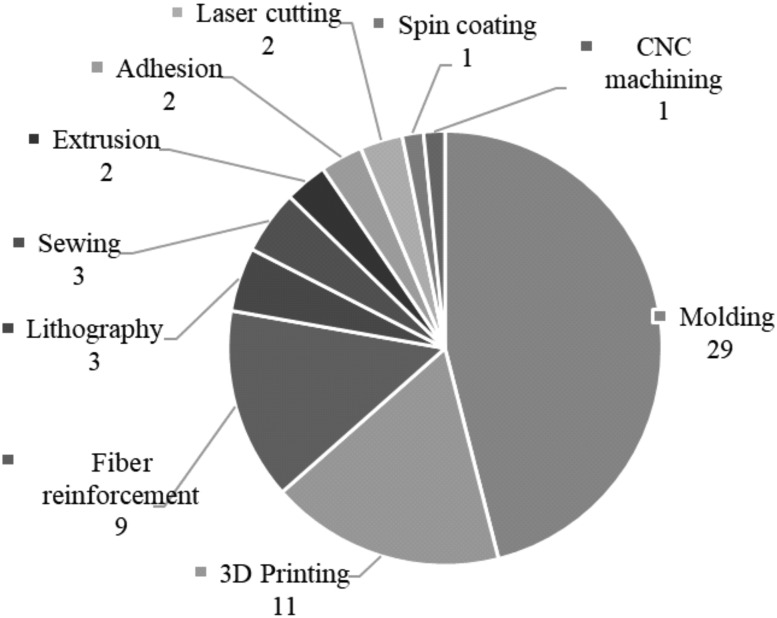
Number of uses of different manufacturing techniques among the selected devices at the focus of this review. More than one technique could be used per device.

Molding of elastomeric polymers was the most popular manufacturing method, possibly because it is a simple process, including the fact that automated processes can be relied upon for fabrication of molds. Figure 3 shows the number of instances where different manufacturing methods were used to make the devices from the literature search, where multiple processes could be used on one device. In most cases, a 3D printer was used to produce the mold, although molds have been made from many materials, including laser cut Plexiglas,^43^ 3D printed plastics, and CNC milled metals. The elastomeric materials that can be used with molding processes are typically soft, making them desirable in minimally invasive applications.

Manufacturing methods such as molding, extrusion, and shape deposition prevent adaptability due to the necessity to newly fabricate or assemble not only the constituent parts but also auxiliary parts that are used in the manufacturing steps, for example, molds or dies that need to be changed to produce a new shape. In contrast, devices can be more readily adapted when they rely on rapid manufacturing techniques that are very easily controlled, taking advantage of computer-assisted design and automated processes, for example additive manufacturing or photolithography.

Difficulties with molding elastomers include bubbles forming in the material before it sets, giving rise to structural inconsistencies. Manufacturing methods were the main problem in the fabrication of a low-cost elastomer endoscope.^24^ The difficulty of demolding larger or more complex shapes can result in damage to the molded part and may require more complex molds. It has also been reported that achieving even thicknesses can be difficult, which is one of the issues caused by using 3D printed molds, as well as the ability to produce small enough molds and prevent them from melting during heating to cure the elastomer.^25^

**FIG. 4.**
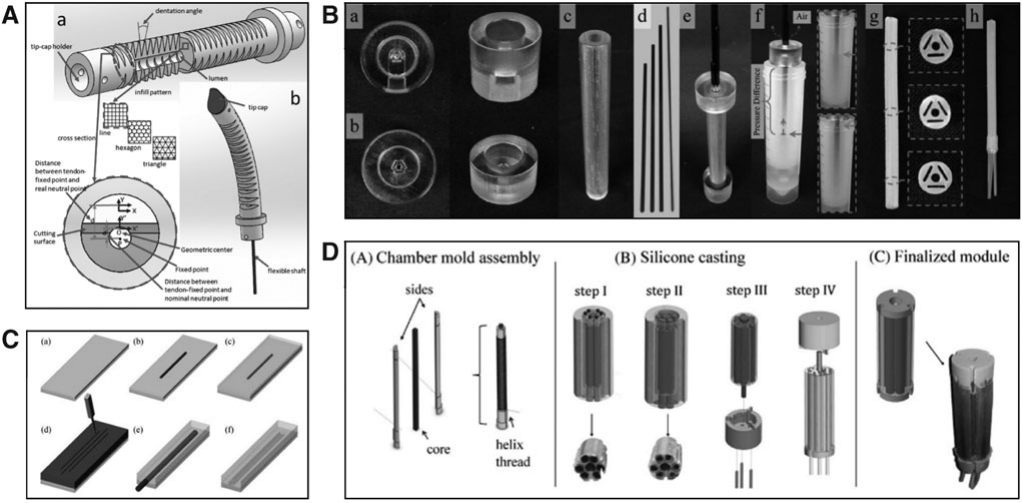
Examples of different manufacturing techniques: **(A)** 3D printing of a continuum manipulator using thermoplastic polyurethane, with different possible infill patterns,^38^ **(B)** stages of an “inverse flow injection” molding method,^28^ **(C)** a microactuator manufactured by multistage spin coating,^34^ **(D)** the steps followed to manufacture a STIFF-FLOP manipulator.^41^ 3D, three dimensional. Figure panels were reprinted with permission from their respective references.

Often, multistage processes are used that can be time-consuming and can introduce errors. These additional steps may include the following: fiber reinforcement, filling with granular particles, inserting layer jamming material, adhesion, sewing, heat crimping, or heat sealing. The review in Gorissen *et al*.^66^ is very thorough in detailing the manufacture of pneumatic elastomer actuators and reports that bonding or sealing elastic materials to create an inflatable chamber frequently leads to actuator failure. Figure 4 highlights a number of manufacturing methods and some of the steps involved.

Using an extrusion process, a helical braided tube device design was made to be adaptable, in that new devices could be made specifically for given pipe diameters. This particular design, however, encountered difficulty propelling itself through pipes with large changes in diameter.^55^ Despite an easily alterable design, the device itself was unable to deal with an adaptable environment, making it unsuitable for MIS applications.

A microscale soft actuator was manufactured by using the spin coating technique to produce a pneumatic channel within multiple layers of PDMS material.^34^ Bending motion was produced upon pressurization due to the asymmetric cross-section of the actuator, which was controlled by precisely adjusting the layer thicknesses of the PDMS. Furthermore, the bending behavior was altered by cutting the profile of the actuator in different ways, for, example, in a trapezium with the pneumatic channel located centrally, or in a rectangular shape with the pneumatic channel placed parallel to the sides at varying distances or at an angle to the sides. These various profiles and channel placements produced a variety of bending behaviors, including curling, spiraling, and bidirectional spiraling.

#### Mechanical programming/embodied intelligence

**FIG. 5.**
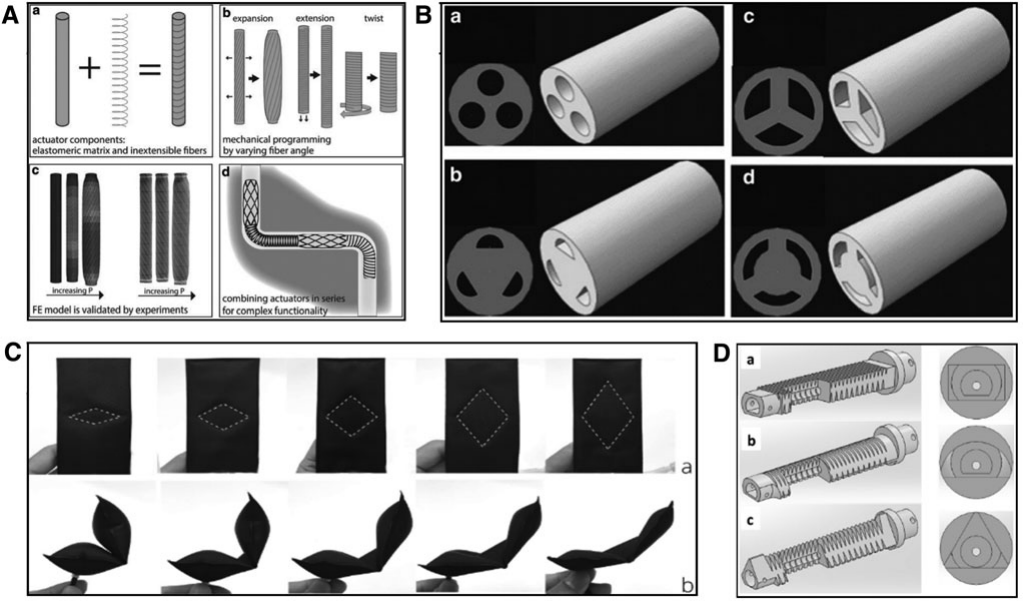
Mechanical programming achieved by varying device design or manufacture: **(A)** a pneumatic actuator made to expand, extend, or twist by changing its fiber winding angle,^67^ **(B)** four variations of a continuum robot body whose inner chambers determine its behavior,^32^ **(C)** different bending angles achieved at a given operating pressure by changing the aspect ratio of a diamond hinge pattern,^68^ **(D)** various cross-sectional shapes of 3D printed manipulators change their mechanical behavior.^38^ Figure panels were reprinted with permission from their respective references.

How a device or component moves is based on its mechanical construction; hence, the designer can alter the behavior of a device by subtly changing how it is manufactured, while maintaining the underlying core design. The area of soft robotics is ripe for this design methodology because behavior during actuation is dependent on the body of the device in many cases. Therefore, there is opportunity to use their construction to influence their deformation. Figure 5 features a selection of soft robotic devices that use this approach.

As an example, the deformation of a pressurized elastomeric fluidic actuator can be constrained by wrapping the actuator in reinforcing fiber. A variety of bending, twisting, expansion, and elongation behavior can then be produced depending on the fiber winding angle without changing the elastomeric actuator. This concept was explored in detail in Connolly *et al*.,^67^ where a self-propelling soft device was manufactured by chaining fiber-reinforced pneumatic actuators in series that were identical, except for the fiber winding angle and the number of fiber layers. Three different fiber winding patterns were used, which resulted in extension, expansion, or twisting of the actuators when pressurized, so a robot capable of peristalsis and with a twisting end effector was produced by connecting and pressurizing the actuators in a specific sequence. This shows how the motion of a simple pneumatic element can be changed by processing it in different ways.

Pairs of finger-like pneumatic actuators of semicircular profile with fiber reinforcement at distinct angles have been coupled together in parallel back to back to produce both bending and twisting motions; however, the bending behavior is asymmetric as a result of the asymmetric design.^25^ Again, by changing the fiber winding angle, the motion of the manipulator can be programmed.

A cable-driven continuum robot was manufactured by 3D printing using thermoplastic elastomer^38^ and the mechanical behavior of the device was shown to change with its cross-sectional shape. Devices with triangular, semicircle, and rectangular cross-sections were manufactured and it was found that the device with triangular cross-section could transmit less force that the other versions. This also demonstrates how using an automated manufacturing process facilitates design adaptations. Various cross-sectional shapes of the actuation chambers, chamber length to module length ratios, and chamber proximity to the outer wall were tested in a pneumatically driven elastomeric continuum device,^32^ where both the pressure required to achieve a 90° bend and the ballooning area were measured. This information could be used to design a manipulator with specific bending behavior.

Pneumatic chambers can be formed by heat-sealing multiple layers of flexible planar materials and complex, actuating structures can be produced by controlling the heat-sealed patterns.^68^ Using this method, the bending angles of pneumatic hinges at a given working pressure were programmed by changing the aspect ratio of the heat-sealed, diamond-shaped pattern.

### Actuation, stiffness variation, and locomotion methods

In this section, three important aspects of soft robotics are discussed: actuation, variable stiffness, and locomotion. These are highlighted because they can instill robotic devices with many of the abilities necessary in medical applications, for example, manipulating objects, supporting loads, and propelling themselves. Advances in actuation, variable stiffness, and locomotion will enable robotic devices that increase surgeon dexterity, access, and the ability to intuitively use their skills as if they were performing an open surgery.

#### Actuation methods

Actuation methods produce motion of a robotic device, permitting bending, elongation, and force exertion, for example. These technologies, appropriately controlled, will give soft robotic devices the dexterity to allow surgeons to perform surgical tasks more easily. Table 5 briefly summarizes actuation methods that were used in the articles resulting from the keyword search, commonly used in soft material robotics.

**Table 5.** Summary of Actuation Methods

*Actuation method*	*Description*	*Advantages*	*Disadvantages*
Pneumatic	Vary pressure of gas within a chamber	Capable of producing a range of movements, for example, elongation, expansion, twisting, and bending, plus stiffening; large volume changes are possible; quick response; very lightweight; good power density.	High pressures needed to exert larger forces; nonlinear response makes control more complex; depends on fluid flow that introduces a delay; risk of puncture or gas escape that could cause damage/harm.
Hydraulic	Vary pressure of liquid within a chamber	Capable of exerting large forces; incompressible actuation fluid makes precise control more achievable; high power density.	Adds weight to device; slower response time; depends on fluid flow that introduces a delay; jets of pressurized liquid could damage soft tissue.
Cable driven	Change tension or length of cable to exert force	Capable of exerting larger forces; fast response; small form factor; lightweight.	Only capable of actuating in one direction, when cable is in tension (zero compression stiffness); friction forces can become large in long cables along tortuous paths.
SMA	Deform alloy by changing its temperature	Easily controlled; medium velocity response.	Sensitive to environment; potential safety risk posed by thermal dissipation; hysteresis error.

**FIG. 6.**
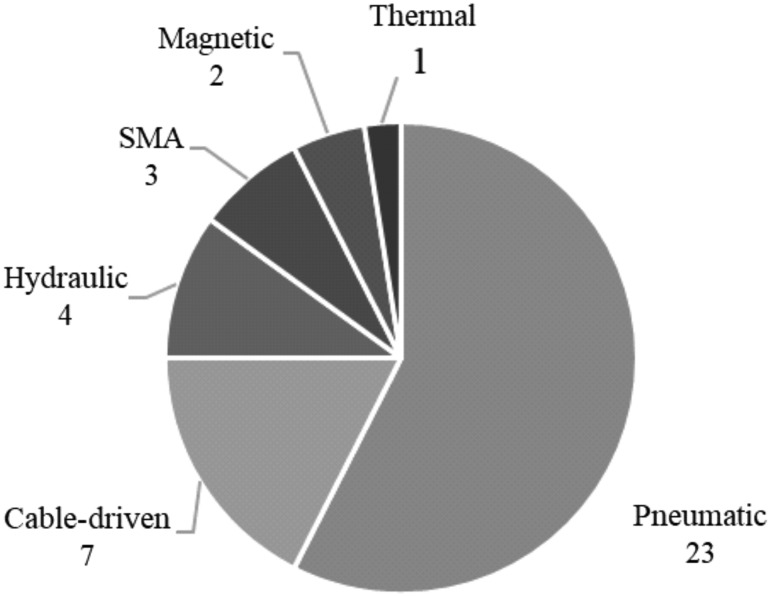
The number of devices from the literature search that used each actuation method, with more than one actuation method being used in some cases.

The most common actuation method used among the devices from the literature search was pneumatic actuation, followed by cable-driven, hydraulic, shape memory alloy (SMA) and thermal actuation. Electroactive polymers (EAPs) are at the center of much research; however, no device using EAPs was present in the results of this literature search. The lack of “dry” EAPs may be attributed to the high voltages that are required for actuation,^69^ and lack of “wet” EAPs may be because only low forces can be exerted and they have long response times.^70^ Figure 6 shows the number of times different methods were used among the results, where multiple actuation techniques could be employed in a single device. The following section describes some examples of the different actuation methods.

Fluidic actuation blurs the lines between actuation and variable stiffness because the internal pressure of a chamber influences the stiffness and will do so in different ways for different actuation fluids. When deformations in the cross-section of a beam occur, this can cause an increase in stiffness due to Poisson's effect.^31^ The coupling of actuation and stiffening effects is a problem for the most popular soft robotic actuation method because the stiffness varies with the pose, making control difficult.

Most devices that use pneumatic actuation are manufactured by molding elastomeric materials and most are continuum robots. Many of these require strain-limiting layers to avoid ballooning. If larger diameter/stronger/stiffer devices are required, then the central channel may be unavailable for instrumentation because it may disturb the pneumatic actuation channel. If a continuum robot is manufactured with instrumentation channels, making it a more useful device in a surgical context, then the trade-off is lower force exertion and larger size because space for actuation chambers is reduced. For highly elastic pneumatically actuated designs, the cross-sectional area, actuation chamber length and wall thickness can change the bending behavior.^32^ Hence, it can be challenging to introduce flexible cameras and instrumentation channels that would normally be found on a traditional endoscope without altering the controllability, the size, or both.

The load-bearing limits of a cable-driven system can be increased by increasing the diameter of the cable, which will not significantly change the size of the device. However, to exert larger forces, the support structure for the cables must be stronger. This may consequently impact on the materials used or the size of the resulting device. Cable- and SMA-driven systems depend upon the structure that they are built into, while fluidic actuators depend upon the materials that constitute the fluid chambers.

There are also safety issues that need to be confronted when using each of these actuation methods, given the sensitive surroundings and the possibility of the surgical instruments interacting with the soft robot itself. The risk of piercing a pressurized chamber can be mitigated by utilizing a protective sheath; however, this may increase size or manufacturing complexity. Another factor to take into account is that the temperature reached by some SMA actuators can exceed the working temperatures of some elastomers.^37^ There are also safety issues related to exposing soft tissue to higher temperatures.

The length of SMA wires affects their mechanical behavior. In Shim *et al*.,^37^ the length of the NiTi SMA wire influenced both the maximum twisting angle and the maximum twisting torque. Similarly, the size of fluidic actuators influences their force exertion capabilities and their rigidity. These examples highlight the trade-off between size and power.

#### Variable stiffness mechanisms

Only granular/particle and layer jamming offer variable stiffness without producing motion, as opposed to actuation methods that can not only cause changes in stiffness but also exert force, for example, cable-driven or pneumatic systems. Granular and layer jamming are compared in Table 6. For a full review of variable stiffness mechanisms used in medical devices, the reader is directed to Blanc *et al*.^71^

**Table 6.** Summary of Stiffening Mechanisms Used in Articles from Literature Search

*Stiffening method*	*Description*	*Advantages*	*Disadvantages*
Granular/particle jamming	Force particles of a granular solid together to stiffen using particle locking	Can achieve large variation in stiffness; shape locking	Adds weight and volume to the design; underlying physical relations unclear, difficult to model; particles can spread unevenly, leading to nonuniform stiffness
Layer jamming	Force layers of a planar material together to stiffen using interlayer friction	Low profile; capable of shape locking; even stiffness if layers are well constrained relative to each other	Adds rigidity in inactivated state; limited increase in rigidity

**FIG. 7.**
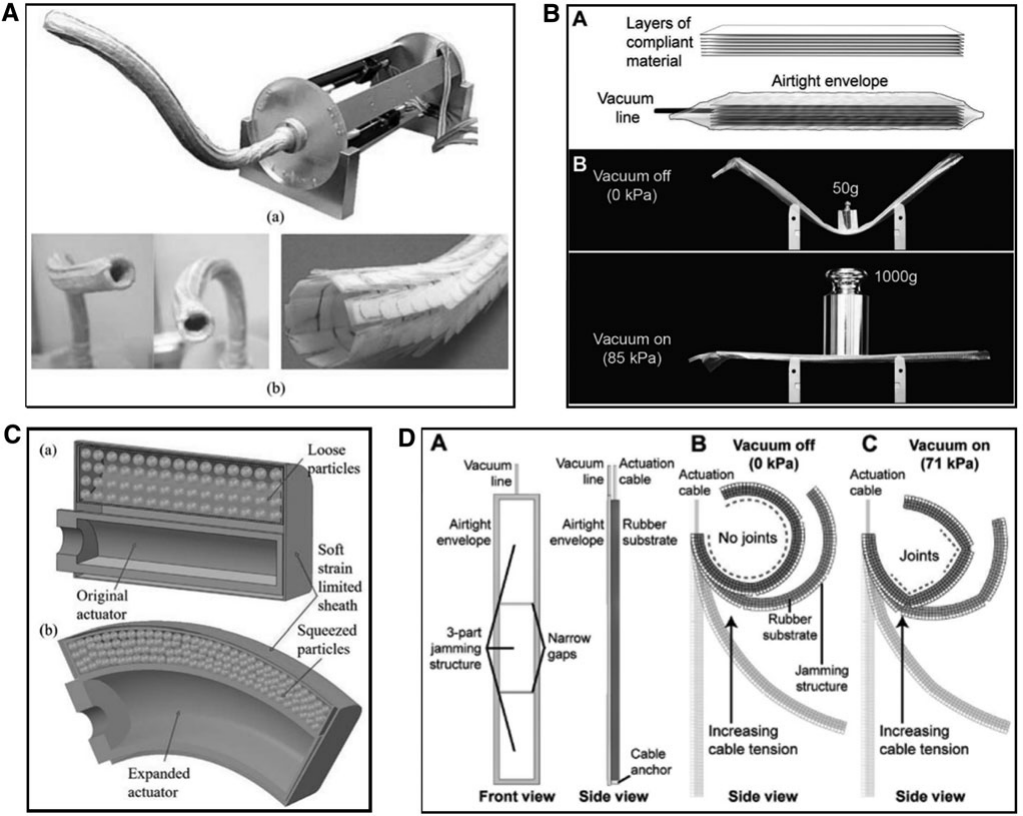
Granular and layer jamming techniques: **(A)** layer jamming employed in a stiffening sleeve for use with endoscopic devices,^21^ **(B)** the layer jamming principle, variable stiffness can be achieved by applying a vacuum to a sealed chamber,^72^ **(C)** a passive particle jamming method to simultaneously actuate and stiffen a device,^35^ **(D)** layer jamming in a cable actuated finger used to alter its structure online.^72^ Figure panels were reprinted with permission from their respective references.

Jamming relies upon locking together the particles of granular materials or several layers of planar materials, which can be induced when pressure is exerted on these materials in enclosed chambers. Figure 7 displays some interesting implementations of jamming techniques. Jamming based on negative gauge pressure is limited by the ambient pressure surrounding the jamming chamber, but can also be achieved by applying a positive gauge pressure to the jamming material. In Li *et al*.,^35^ jamming with positive pressure is achieved by constraining a finger-like elastomer device consisting of both an expanding pneumatically actuated chamber and a jamming chamber filled with 2 mm diameter glass beads within an inextensible leather sheath. Inflating the pneumatic chamber causes it to expand radially, pressing the jamming chamber against the outer sheath and locking the glass beads together to increase stiffness. This design increases in stiffness as it bends and uses only one actively actuated chamber; therefore, it is a useful configuration in applications where the number of input tubes or cables linking a device to its power source needs to be minimized, or the design needs to be as simple or robust as possible. In general, a high number of jamming layers, high actuation pressure, and high coefficient of friction of the laminar material are recommended to achieve the highest stiffness change using layer jamming.^72^

Several actuation methods can be used together to produce an increase in stiffness, for example, pneumatic actuation and a cable-driven system as in Stilli *et al*.^22^ and Shiva *et al*.^39^ Actuation and variable stiffness mechanisms can be used together, for example, pneumatic actuation and granular jamming as in Li *et al*.,^35^ Diodato *et al*.,^44^ and Cianchetti *et al*.^45^ or a cable-driven system used together with a layer jamming mechanism as in Kim *et al*.^21^

The combination of variable stiffness mechanisms and soft, flexible materials has been used to alter a robot's kinematics online, something that is impossible with traditional, rigid robots. A two-finger, elastomeric cable-driven gripper featuring three serially connected layer jamming modules per finger was manufactured in Narang *et al*.^72^ and was able to move each finger either as a continuum mechanism, when the modules were unjammed, or as a serial mechanism with two joints between the three jammed links.

#### Locomotion

**FIG. 8.**
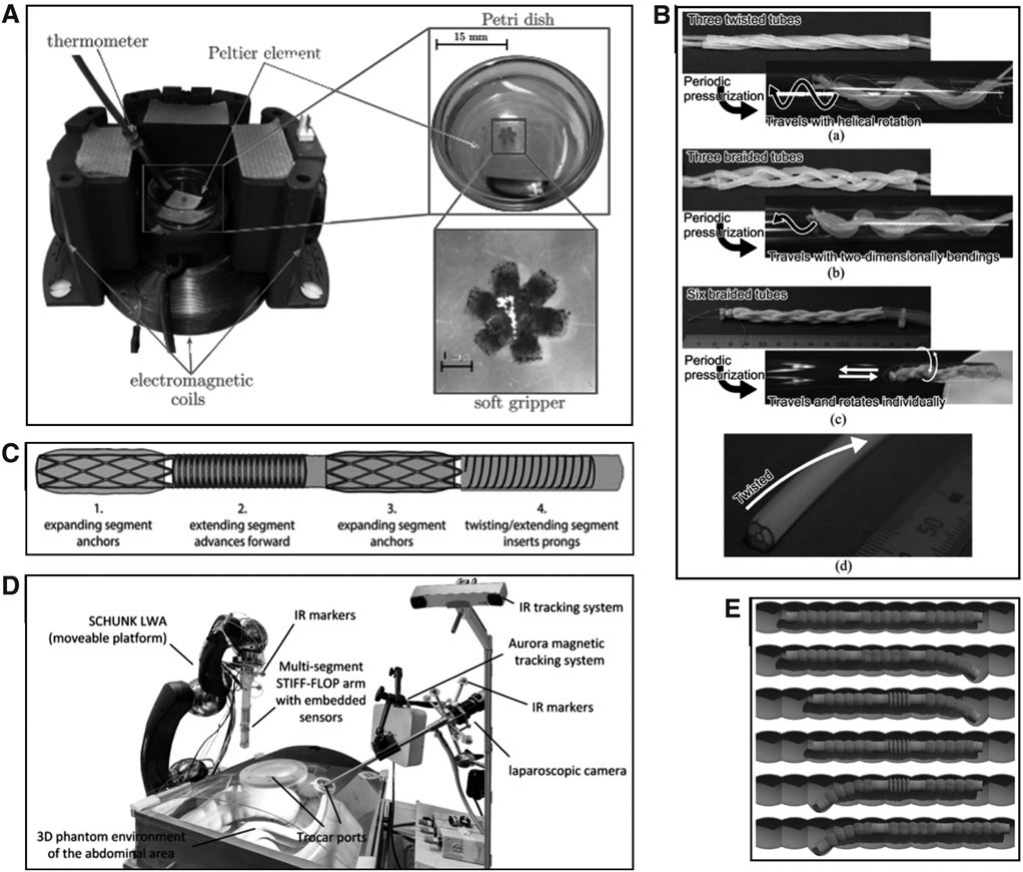
Locomotion methods: **(A)** magnetic fields used to maneuver a microgripper on a Petri dish,^50^ **(B)** braided actuator bundles achieving both peristaltic forward motion and twisting in different configurations,^55^ **(C)** multiple pneumatic actuators each used for different parts of a peristaltic locomotion cycle,^67^ **(D)** a robotically guided STIFF-FLOP manipulator,^42^ **(E)** visualization of an inchworm-style crawling robot anchoring on the inner colon wall by applying a force.^46^ Figure panels were reprinted with permission from their respective references.

Robotic locomotion in the context of medical devices should aim to enable access to areas that are difficult to reach and do so without causing patient discomfort. At present, the most common method of delivering a robotic device to the target tissue is by manual insertion, which is a problem for devices made of nonrigid materials that will buckle under loading, and also for those constructed from rigid materials that are difficult to pass through the tortuous paths of lumen within the body without stretching tissue and causing pain. Another method is robotic guidance, where a soft robotic device may be attached to another robotic device, typically a rigid device, which controls the soft device's positioning.^20,44^ Magnetic fields can also be controlled to precisely position micro robots that could be used for drug delivery,^49,50^ although the hardware needed to achieve this can be bulky. Finally, self-propelling devices use on-board mechanisms to interact directly with their surroundings to exert thrust forces. Peristaltic crawling was the only self-propulsion method developed in the results of the literature search. Figure 8 highlights a number of locomotion methods revealed by the literature search. For an in-depth analysis of soft robotic locomotion, the review^54^ is a valuable resource. Table 7 briefly summarizes two types of peristaltic crawling, inspired by the earthworm and inchworm, respectively.

**Table 7.** Summary of Peristaltic Crawling Types

*Locomotion method*	*Description*	*Advantages*	*Disadvantages*
Earthworm-inspired peristalsis (wave)	Waves of anchoring and elongation pass along whole body	Higher friction may be generated by a greater number of anchoring sections; radial force exertion, may prevent looping.	Multiple actuation units to be sequentially controlled increases controller size and complexity; no existing sensors for speed or position measurement.
Inchworm-inspired peristalsis (two anchor)	The head and tail are sequentially anchored, while the body elongates and contracts	Simple sequential control is possible; radial force exertion, may prevent looping; shorter body than earthworm style.	Movement is jerky; no existing sensors for speed or position measurement.

**FIG. 9.**
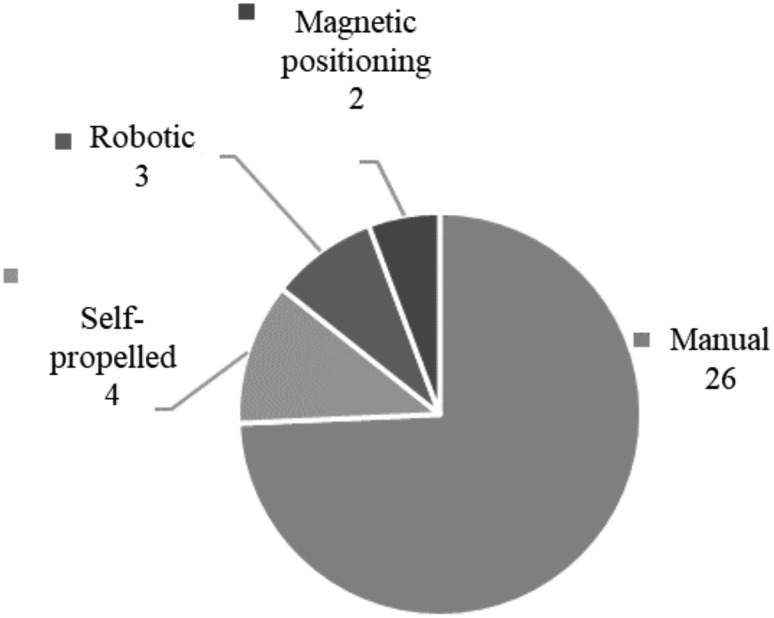
Locomotion methods of devices from the literature search.

Figure 9 shows the reliance of the devices yielded in the literature search on the method of manually placing the device, a method that plays a large role in causing patient pain. Patient discomfort is one of the main issues with colonoscopies currently,^73^ so a reliable and painless self-propulsion method would be of great value to the field.

The method proposed in Bernth *et al*.^46^ shows the ability to self-propel using a two-anchor inchworm method; however, it relies upon anchoring at a single point on the colon wall each cycle and this still may cause stretching and pain to the patient. Another way may be to anchor the device across a larger area so as to distribute the load, and this could be achieved by using more compliant materials that would deform to the inner colon wall.

A specifically designed anchoring mechanism was developed on the design of an endoscope attachment in Stopforth *et al*.,^24^ with the goal of realizing autonomous exploration of the colon. The hydraulically powered endoscope probe could align itself with the center of a tube, and an image processing system designed to find the center of the colon was developed.

The existing soft self-propulsion mechanisms of the devices in the literature search are still slow. The highest speed of the peristaltic crawling device in Bernth *et al.*^46^ is 1.21 mm/s, with the authors stating it could travel from one end of a human colon measuring 1850 mm^74^ to the other in ∼30 min. It takes between 20 min and 1 h to complete a standard colonoscopy,^75^ so the device would offer no improvement on procedure time. The friction force in the anchored state of Bernth *et al.*^46^ was 2 N.

Using a different approach, three designs of three or six elastomer pneumatic tubes, either braided or twisted together, were developed in Takeshima and Takayama.^55^ The twisted three-tube device moved through a pipe with a helical trajectory, the braided three-tube device moved side to side in a serpentine manner, while the braided six-tube design could translate and rotate independently about the axis of the pipe. However, none of these were successful moving in pipes that had changes in diameter. A trade-off between locomotion capabilities and size must be confronted with peristaltic designs, as there, the physical extension of the device is relied upon. For example, the forward thrust force of the device in Takeshima and Takayama^55^ is dependent on its length. For applications within the lower GI tract, the problem of lack of traction in the unsupported, elastic colon still has no definitive answer.

A greater focus on locomotion methods for soft robotic devices in the lumen of the body will also enable NOTES, further reducing recovery times and eliminating visible scars. A problem with NOTES in terms of design of a self-propelling device is that a change in locomotion method will likely be required as the device transitions between tubular structures such as the GI tract to the less constrained abdominal cavity. However, soft material robots are capable of changing their structure, volume, and working principle, among other qualities, so they are an appropriate candidate technology for solving this issue.

### Sensors

This section describes different categories of sensors and how they might be used to provide useful information in the context of MIS. For a review of soft electronics, the reader is directed to Dickey.^76^

To gain meaningful knowledge about a robotic device, the environment that surrounds it, and the interaction between the two, measurements from a variety of sources must be taken. Sensors can be grouped into two main categories: proprioceptive and exteroceptive, which, respectively, measure characteristics of the robot itself and characteristics of its surroundings. Sensors of both types are important for control strategies and, in the MIS context, for preventing the robot from causing harm to the patient, for example, by exerting large forces on soft tissue or cutting off blood supply through prolonged pressure exertion and causing ischemia. Diagnostic sensors also gather information, not pertaining to robotic devices, but rather to provide valuable medical information about the patient.

**FIG. 10.**
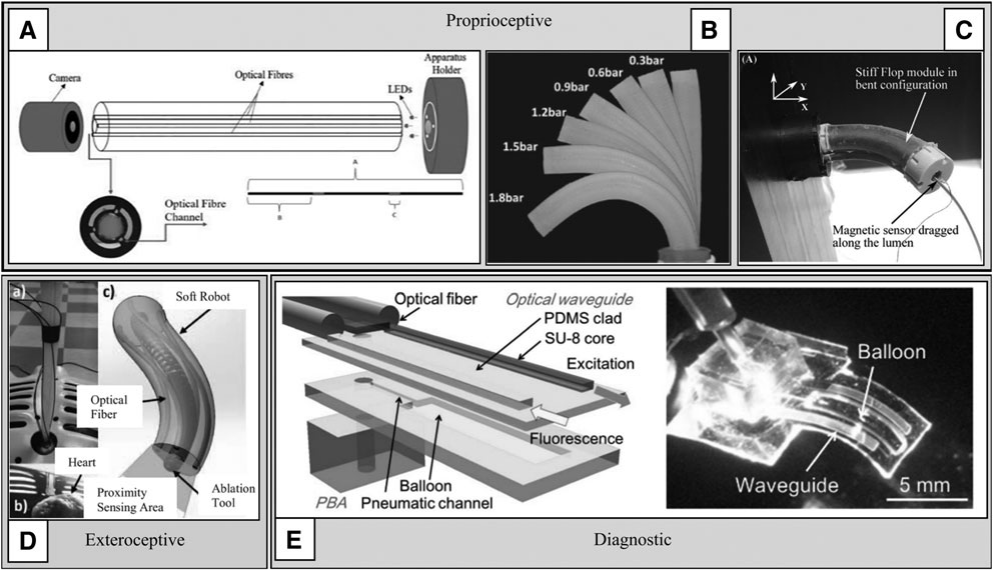
Proprioceptive, exteroceptive, and diagnostic sensors in soft robots: **(A)** bending angle estimation using light intensity transmitted by optical fibers,^79^ **(B)** using interior air pressure to estimate angle,^29^ **(C)** using an EM tracker to record the pose of a STIFF-FLOP manipulator,^41^ **(D)** optical fibers for proximity sensing integrated into a cable-driven cardiac ablation tool,^19^ **(E)** an optical waveguide for fluorescence imaging integrated into a miniature elastomer actuator.^30^ EM, electromagnetic. Figure panels were reprinted with permission from their respective references.

Often the information gathered by one category of sensor is useless without information from another, so fusion of information from proprioceptive, exteroceptive, and diagnostic sensors is often required. In many cases, it is the output from diagnostic sensors that must be coupled with either proprioceptive or exteroceptive data to deliver useful information. A simple example could be a device that can detect early signs of cancer in the GI tract, which is made much more useful by also being able to accurately establish where the device, and therefore the cancer is located, for subsequent retargeting. Figure 10 displays some approaches to proprioceptive, exteroceptive, and diagnostic sensing with soft robots that are discussed further in this study.

Difficulty modeling soft robotic devices is a substantial problem. For sensor data to be used appropriately in a range of applications like control, haptic feedback, or diagnostics, a model of the robot in question is required. This poses a great challenge in the field of soft robotics because of the nonlinear behavior of many soft materials and actuation methods.^77^ Therefore, extra consideration must be given to sensor design, placement, and data interpretation. Furthermore, for shape changing soft robots that can reversibly transition between different kinematic regimes, numerous models may be needed to fully describe them. For example, the number and location of bending angle sensors on a continuum robot will differ from that of a serial robot, and the information the sensors provide needs to be contextualized appropriately in each case.

Empirical performance modeling was necessary in Takeshima and Takayama^55^ due to the difficulty of computationally modeling the braided three-chamber pneumatic device that had been developed. This highlights the need for less expensive computational modeling of soft robotic devices to achieve the aim of developing more accurate control systems without the need for experimental characterization of each individual device, which would inhibit customizable, patient-specific robots, for example.

#### Proprioceptive sensors

In the results of the literature search, there were more examples of proprioceptive sensors than exteroceptive or diagnostic sensors. Proprioceptive sensors are often used for control of robotic mechanisms because they can be used to estimate the pose or shape of the device, hence their high priority. There are a broad range of sensors that can be used for proprioception of soft robotic devices that do not significantly increase the stiffness, and they vary depending on the working principles or actuation methods that are being employed. Robots that are actuated using fluids may include pressure sensors,^23,24,39,40^ although there is often difficulty relating internal pressure to bending angle, especially in the presence of external forces on the device.

Cable-driven robots have used measurements of cable tension^39^ and length, for example, using Hall effect sensors,^46^ but effects such as cable friction can affect these results. Robots made of elastomeric materials may use strain gauges to estimate the shape of the device, although none of the devices in the literature search used these. The field of soft strain sensors is well established because of their biomedical and wearable applications, and Amjadi *et al*.^78^ is a useful review on the subject.

Optical fibers have been used with soft continuum robots to sense their bending angle. In Al Jaber and Althoefer^79^ three optical fibers were placed in a deformable rubber cylinder at 120° to each other to estimate the curvature. A camera was attached to the end of the cylinder to detect the light transmitted through the optical fibers, emitted by RGB LEDs. By monitoring the intensity of the light in the camera feed, as well as the size, orientation, and position of the optical fiber tips, the bending angle could be estimated with an error between 2.3% and 17.6%. This method is promising; however, it requires bulky hardware that would alter the mechanical behavior of a soft robotic device. Fiber Bragg Gratings (FBGs) embedded in optical fibers were integrated into a cable-driven soft continuum manipulator for the purpose of shape sensing in Wang *et al*.^80^ Despite showing good performance for simple robot configurations, it showed sensitivity to temperature change, inaccuracies in molding manufacture affecting optical fiber placement, and insensitivity to large deflections over short lengths of the soft robot.

Electromagnetic (EM) sensors have been used to track the tip of continuum robots,^29,32,38,43^ but these measurements can be affected by stray EM fields from other equipment^81^ or field distortion in the presence of metallic structures. Using continuum soft robots equipped with EM sensors, machine learning has been used to control tip position along given trajectories, which in each case improved the robots' abilities to follow a path.^29,82^ It would be valuable to see results of this approach using other sensor inputs that do not suffer from the same issues as EM trackers.

Imaging methods such as fluoroscopy and ultrasound can be used for shape sensing; however, fluoroscopy requires costly, bulky equipment and exposure of the patient to radiation, while ultrasound offers only low resolution.^81^

A novel additive manufacturing method has been developed to simultaneously print elastomer and liquid metal in a helical structure to form an inductive sensor that is sensitive to tensile and bending deformation.^83^ This flexible helical sheath is designed to be placed around a soft, cylindrical device such that the bending angle of the device and the sensor is the same. Deformations in the sensor then cause changes in inductance of the liquid metal core, which in this case is galinstan, an alloy of gallium, indium, and tin. Using this sensor, the bending radius of an endoscope was estimated in distinct poses with an error of between 1.53% and 7.96%. Using liquid metals or other fluids as sensors permits highly flexibility and compatibility with soft robots; however, liquid metals can be difficult to handle and will add weight and bulk to a robot or sensor. Finally, liquid metals such as galinstan and eGaIn, a eutectic mixture of gallium and indium, create increased safety concerns as they are not biocompatible.^84^

#### Exteroceptive sensors

Exteroceptive sensors may be cameras, force sensors, or contact sensors and it is this class of sensor that could provide benefits to the surgeon such as force feedback or positioning and orientation information relative to the surrounding tissue. In the literature search results, there were only two examples of integrated exteroceptive sensing. One of these, a cable-driven, continuum robot designed for cardiac ablation, was manufactured with four optical fibers integrated into its elastomer body, which could measure the distance of the tip to a beating heart phantom.^19^ This proximity sensor was shown to reduce the error between the desired and actual distance from the distal end of the robot to the beating heart model, thereby facilitating the ablation procedure.

A method for estimating the normal and shear forces exerted on a hydraulically actuated STIFF-FLOP manipulator^18^ by developing a nonlinear finite element model to relate tip deflection and internal pressure change is given in Lindenroth *et al*.^23^ Maximum normal force errors of between 0.2 and 0.4 N for bending angles between 0° and 60° were reported, while maximum shear force error was dependent on the shear load itself and the tip deflection, with a maximum error of 0.316 N for low tip angles and a load of 1.5 N. The hardware used for pressure measurement does not introduce rigid components to the manipulator, so the robot can still be deemed soft. When large shear forces were exerted on the robot, the force estimation errors increased; hence, further research is needed to develop more robust force sensing abilities. Developing a soft approach to force sensing that avoids bulky hardware is a promising target for research, which could provide soft robot operators with the sense of touch in the future.

#### Diagnostic sensors

A number of devices described in articles from the literature search included integrated cameras that would allow visual inspection of the immediate surroundings; however, only one device featured dedicated diagnostic equipment. The device integrated photoresist material into a pneumatically actuated elastomer that was used as an optical waveguide for fluorescence imaging.^30^ The fluorescence is detected at a given input pressure, which can then be related to the bending angle of the device. This may be impractical if the bending angle of the probe cannot be measured accurately due to interactions with an unstructured, dynamic environment where the probe may come into unintentional contact with its surroundings, resulting in an inaccurately measured location of fluorescence. The inability to measure the bending angle of the soft actuator accurately again highlights the need for proprioceptive, exteroceptive, and diagnostic sensor fusion.

Optical fibers can be integrated into soft robotic devices, so it would be interesting to see some more diagnostic applications of optical fibers in soft robots. This may include optical biopsy techniques such as diffuse reflectance spectroscopy, optical coherence tomography, and endomicroscopy.

## Discussion and Conclusion

### Comparison

There exists no consistent standard of comparison for soft robotic devices designed for minimally invasive procedures. Given the variety of devices and operating principles under consideration, this makes it very difficult to compare candidate devices and identify knowledge gaps, engineering challenges, and improvements. A standardized datasheet is proposed in Grioli *et al*.^85^ for use in defining variable stiffness actuators. The template suggests designers graphically define relationships such as stiffness, speed, and deflection versus torque and the 3D workspace, as well as giving operating data, including output power, nominal and maximum torque, speed, stiffness, elastic energy, and sensor descriptions. In total, a very detailed picture of a variable stiffness actuator can be built.

However, it can be problematic to attempt to describe a device using only limited measurements, especially when the measured quantities are dependent upon each other. This is the case with some pneumatically actuated devices that have incorporated stiffening mechanisms, so the bending angle, force exertion, and stiffness are all interrelated. The pose of some soft robotic devices can also affect their mechanical properties, so it is indeed challenging to meaningfully describe these devices only in numbers. Furthermore, the proposed datasheet is for continuum-style actuators, so other robotic devices cannot be well compared, although the datasheet is quite comprehensive.

Instead, a focus on the capability of the proposed device to carry out a simplified, standardized version of the application it was designed for could be more useful and could make the advantages of the design clearer. An “assault course” of standard tasks, each related to a specific application, that all new devices could be tested on would provide metrics on whether or not a device provides performance advantages in each area. This approach, coupled with a standard datasheet, would make comparison far simpler.

### Application and working principle

Given that continuum robots experience issues balancing size, force exertion, and controllability, there are few examples of alternative approaches in the literature. Continuum and serial robots do appear to be a good choice of manipulator; however, these robots also struggle with access to the target anatomy. Self-propelled devices with integrated continuum or serial manipulators may be the best way to improve on these issues. Providing a mobile base for robotic manipulators will require the development of variable stiffness mechanisms that can withstand higher forces; reliable self-propulsion methods that can both achieve higher speeds and remain minimally invasive; and anchoring methods so that higher forces can be exerted by the device when it is *in situ*.

Designers could also take advantage of soft robotic devices' ability to change shape, volume, and even working principle online. Potential applications of this could be a soft robot to perform NOTES that can move through the GI tract using one locomotion strategy, and then change shape to use a different locomotion strategy in the abdominal cavity.

New soft robotic devices should aim at integrating soft proprioceptive, exteroceptive, and diagnostic sensors. The results of the literature search yielded very few examples of diagnostic sensors and many less well-developed devices did not clearly state what benefits they would deliver to surgeons in terms of what instruments could be used, if any channels were available. Furthermore, few devices described what the effect of having different instruments in place would be on controllability or force exertion. Traditional endoscopes provide a platform to quickly transition from diagnostic to therapeutic operation, for example, during a colonoscopy, a traditional endoscope can be used to not only image the colon but to also perform surgical procedures, although limited. Soft robotic devices for minimally invasive procedures should therefore also aim to provide both diagnostic and therapeutic capabilities. The advantages of traditional endoscopes can be expanded upon and the therapeutic capabilities improved by attaching deployable structures to the endoscope tip, such as the inflatable structure in Vrielink *et al*.,^86^ which supports two cable-driven surgical instruments for intuitive, bimanual control.

As can be seen from the devices found in this study, very few soft robots for minimally invasive applications are made to be deployable. Deployability in this context is the capability to unfold or change shape to transition from a low to high volume, and in medical applications could be very useful for gaining access deeper into the body without the need for incisions to the abdominal cavity. This indicates that there are still important challenges to be overcome relating to the scalability of actuation mechanisms and the robots themselves, and that the material choices of the designers have not permitted deployability.

Almost all the devices require a tether and, in the context of MIS or colonoscopy where there is limited working space, this seems to be a reasoned design choice if the total footprint of the hardware is not extensive. Conversely, the long-term goal for the future should be the development of miniaturized, untethered, soft robots able to achieve unprecedented access throughout the body, causing no damage as they operate. Much work has been done in the field of capsule endoscopy to miniaturize vision and locomotion systems and incorporate them in individual untethered devices. As challenges such as achieving reliable and accurate control and achieving sufficient force exertion from miniaturized actuators are solved, the integration of approaches from capsule endoscopy and the field of soft robotics will become possible.

A soft robotic device is described in Wehner *et al*.,^87^ which can operate autonomously using pneumatic elastomeric actuators powered by on-board chemical decomposition. Questions over the safety of using devices that rely upon chemical reactions in medical applications have been raised, but devices of this nature may become feasible for use in minimally invasive procedures if safety issues can be solved. Furthermore, the question of how much autonomy to give robotics devices within the body will need to be addressed.

### Materials and manufacture

There is very little variety in the materials used in the soft robots encountered by the literature search. Very few designs use films of inextensible materials to make chambers for fluidic actuation and this may be because there does not currently exist a simple manufacturing method that uses these materials, in contrast to elastomers that can be processed by simple molding procedures.

Researchers in this area should explore a larger range of materials, although this may induce higher cost. Maintaining high performance at low cost is the key to making surgical technology accessible. Improved and accessible tools will enable surgeons with less experience to perform more challenging procedures, while low-cost solutions will prevent improved robotic systems from becoming exclusive to certain locations.

A new manufacturing approach would help to overcome the problems currently faced by soft robotics in MIS. Designers of soft robotic devices for MIS should consider manufacturing techniques that are low cost, fast, and capable of producing small features. High repeatability and reliability are also necessary, as well as being able to adapt both the designs and the manufacturing process. High adaptability also applies to being able to add or take away device functionality, for example, sensors or locomotion mechanisms, depending on what is required from the surgical procedure. Design adaptability and economical consumables will also enable disposable patient-specific soft robotics, which may reduce complications.

There may be certification issues regarding patient-specific devices and their route to obtaining CE marking, for example. As part of The Medical Devices Regulations 2002 legislation made by the UK government, a custom-made device is one that is made for the sole use of a particular patient. This legislation states that no custom-made device can be marketed unless the manufacturer can ensure the manufacturing process is capable of producing each individual device up to the given standard for that type of device. This shows the importance of developing devices using highly repeatable, reliable manufacturing processes. This could be problematic for devices that rely on molding processes that are sensitive to manufacturing defects such as bubbling or nonuniform thickness.

### Actuation

None of the actuation methods mentioned are reversible so, for example, a cable or pneumatic actuator can only push or pull, but cannot do both. Using actuators that can only exert forces in one direction has the consequence that creative ways of exploiting material characteristics, combinations of actuators, and antagonistic arrangements of actuators are necessary. This requires more space and increases device complexity, which drives up manufacturing times, costs, and creates a conflict with competing factors such as the need for instrumentation channels.

The demand for actuation methods that are powerful, fast, lightweight, and low volume is also left unmet currently, but this may also be related to the design choices being made. Soft parallel mechanisms have not been explored and may provide improvements on the force exertion and controllability issues common to continuum designs. Manufacturing methods that can produce pressure chambers capable of withstanding much higher actuation pressures would deliver an increase in force exertion, and using both inextensible and elastomeric materials might result in higher maximum rigidity or larger ranges for variable rigidity methods. Difficulty modeling nonlinear systems such as pneumatic chambers and soft elastomer bodies also hinders the ability to design devices that can be controlled predictably.
